# The Critical Role of Coping Strategies in Moderating Loneliness and Quality of Life: Parallel and Unique Processes among Transgender and Heterosexual Cisgender People in Pakistan

**DOI:** 10.3390/ijerph18179109

**Published:** 2021-08-29

**Authors:** Samiya Batool, David L. Rowland

**Affiliations:** 1 Punjab Institute of Mental Health, Lahore 54000, Pakistan; samiya.khalid25@gmail.com; 2 Department of Professional Psychology, Bahria University, Islamabad 44000, Pakistan; 3 Department of Psychology, Valparaiso University, Valparaiso, IN 46383, USA

**Keywords:** transgender, coping strategies, loneliness, social support, quality of life, cisgender, psychological health, social relations, physical health

## Abstract

Groups marginalized and/or isolated by minority status—including transgender individuals—are at significant risk for loneliness and diminished quality of life (QoL), effects that can be mitigated to some extent by coping styles. In this study, we examined the relationships among coping styles, loneliness, and QoL outcomes in a marginalized but understudied gender minority group, namely, 200 transgender individuals living in communities in an emerging/developing non-Western geo-cultural region of South-Central Asia (Pakistan), comparing them against a reference group of 100 heterosexual cisgender individuals. Results indicated strong relationships among coping styles, loneliness, and QoL in both transgender and cisgender groups. Moderating variable analysis revealed that coping skills—whether adaptive or maladaptive—help explain differences in loneliness and QoL not only between trans- and cis-gender individuals, but also within just the transgender group. The implications of these findings for intervention strategies to improve QoL among transgender populations are discussed, with reference to both the specific context within Pakistan and the larger context of transgender marginalization within many developing/emerging countries.

## 1. Introduction

Vulnerable groups, including the aged and those marked by minority status related to race, disability, religion, and gender/sexual identity, are often marginalized and isolated from mainstream society. Paramount to the effects of chronic marginalization and isolation are feelings of loneliness, characterized by the sense of being disconnected from others and/or lowered social care [1,2,3]. Both physical and social separation/isolation can disrupt ongoing relationships and communal networks, weakening social ties and precipitating loneliness as new ties remain undeveloped [4,5]. Even in the absence of physical and social separation, loneliness may result from an unfriendly environment that lacks affectionate care and help, with family rejection and negligence, for example, being major predictors of risk behaviors and depression [6]. Furthermore, perceived dissimilarity with peers may exacerbate feelings of loneliness, with those who differ from the majority in terms of race, nationality, religion, physical parameters, or sexual/gender identities being marginalized [7]. Finally, the very outcomes of loneliness—depression, anxiety, and high-risk behaviors—may worsen the situation, resulting in behaviors that further isolate the individual from peers and social networks [8].

### 1.1. Outcomes of Loneliness and Effects on Quality of Life (QoL)

Loneliness is linked to multiple negative outcomes, including poorer psychological and physical well-being [9]. Negative psychological outcomes include distress, depression, anxiety, hostility/anger, impaired emotional self-regulation, and suicidal ideation and behavior [1,2,10,11,12]. Loneliness has also been linked to diminished physical health, presumably through multiple pathways: for example, by dint of the person’s status (e.g., limited access to health care or housing), through risky behaviors (e.g., drug use or sex work: Ref. [13]), and through the added stress on somatic functioning [14,15,16]. Given such outcomes, overall quality of life (QoL)—that is, one’s perception of their position in life in the context of the culture and value systems in which they live and in relation to their goals, expectations, standards, and concerns [17]—is significantly diminished in individuals indicating high levels of loneliness [8,18].

### 1.2. Coping Strategies and Loneliness

Loneliness and feelings of isolation often lead to coping behaviors aimed at mitigating these negative feelings. Coping behaviors attempt to manage the stressful situation, either by changing the parameters of the ongoing situation or by actively managing the emotional stress related to the situation [19]. Coping strategies may be adaptive or maladaptive. Adaptive strategies include such actions as problem solving (e.g., to remove the stressor or oneself from the stressful situation), seeking social support, positive reframing, and relaxation techniques; and the use of such strategies has been linked to better psychological health and self-esteem, greater emotional regulation, and lower depression [20]. Maladaptive strategies may include denial, avoidance, self/other blaming, social withdrawal, substance abuse, aggression, and escape. In many instances, maladaptive strategies are associated with high levels of distress due to situations that seem uncontrollable or unchangeable to the individual [21]. Thus, understanding an individual’s coping strategies for loneliness requires assessment of both adaptive and maladaptive approaches, the former promoting overall psychological and physical well-being, and the latter usually worsening them [22,23].

One positive coping strategy for countering loneliness is finding ways to lessen the gap between the desired and actual level of social relationships, a process that involves taking small initiatives to increase social contact, often supplemented by engaging in healthy and positive activities in spare time [24]. Unfortunately, many of the methods available to the general public for increasing social contact—going to clubs, initiating conversations, joining social/hobby groups—are not viable/practical options for individuals who have been purposefully stigmatized or isolated; for them, establishing new relationships may be particularly daunting.

### 1.3. Transgender Isolation, Loneliness, and QoL

One marginalized group that has been understudied in non-Western geo-cultural regions with respect to psychological health and QoL is that of the transgender population. Social attitudes regarding gender and sexual minorities differ widely across geo-cultural regions, ranging from acceptance and assimilation to marginalization, hostility, and maltreatment [25,26]. In social systems where gender roles are rigidly defined, deviation from binary gender identities may be viewed as pathological and/or as a threat to the cultural norm. As such, the social system may actively discourage or suppress gender non-conformity [27,28,29], and if unsuccessful, the gender-atypical individual may be subjected to stigmatization, marginalization, isolation, and in some instances, physical harm [30,31,32,33,34].

Furthermore, gender and sexual minority individuals experience multiple barriers to accessing resources (e.g., housing, healthcare, education), having not only to overcome heterosexism and discrimination at individual, group, and institutional levels, but also to deal with oft-reported negative experiences particularly when seeking support in those areas. According to the minority stress model [35], the avoidant behavior that results from such negative experiences places minority individuals at further risk, perpetuating a cycle of inadequate care, deteriorating health, and diminished sense of well-being and QoL [36]. Multiple stressors may negatively impact their ability to cope; as a result, their health-protective behaviors diminish and health-averse behaviors increase, placing them at-risk for further victimization and exploitation [37].

Due to their marginalization, transgender persons in many societies face poverty, depression, and lack of access to resources (housing and health care). In addition, they show greater drug use, engage in more risky behaviors tied to their discriminatory status (sleeping in the park), and endorse belief systems that estrange them from mainstream cultural or religious values [38,39,40,41,42]. They, along with other sexual minorities, are at high risk for loneliness, given that their situation meets a disproportionately high number of the predictors for loneliness: physical separation, dissimilarity with peers, rejection by family and friends, changing social status/role, and social stigmatization [43]. The toll on physical, psychological, and social well-being is reflected in the lower QoL typically reported by transgender individuals.

### 1.4. Understanding Transgender Marginalization within Specific Cultural Contexts

Research on the mental health effects of gender minority status in non-Western nations/cultures has emerged in recent years as a public health-related topic, though such initiatives have been limited both in number and in scope (e.g., geo-cultural regions). Yet, a person’s response to the distress of stigmatization and marginalization is greatly influenced by the cultural values and traditions in which the individual is embedded. That is, culture defines acceptable vs. objectionable sociosexual roles and therefore plays a critical role in the way men and women describe, interpret, and ascribe meaning to their specific status [26,44,45,46,47,48].

In Pakistan, a Central Asian country with the second-largest Muslim-majority population in the world, marginalization due to gender minority places heavy psychological burdens on the individual. Transgender people in this geo-cultural region face significant human rights issues [48,49,50,51], although this has not always been the case. Prior to British rule, transgender people (sometimes referred to as “hijra”) had an important role and status in the Asian subcontinent where, for example, they guarded the ladies of the harem and were considered servants of the nobility, often being promoted to key advisory roles [52]. In the historical tradition of the region, they were afforded significant value based on the belief that they blessed people with fertility [53]. Against this tradition, however, in the late 19th century, the British colonized Central Asia, passing morality laws that labeled the hijras as “sodomites” and restricting their activities, inheritance, and other rights [53].

Thus, in the post-colonial era of today, members of sexual/gender minority groups in Pakistan continue to experience social stigmatization and marginalization. To cope with such effects, transgender people often form their own social support systems, “transgender communities,” now found not only in the Asian subcontinent (known as hijra communities), but also in many other parts of the world [53,54,55,56,57,58,59,60]. Specifically in Pakistan, the vast majority of known transgender individuals reside in collective transgender clans [52], fostering a sense of “transgender community belongingness” [61] through both identity and locale. Such communities may confer substantial benefits to their members, with sexual/gender minority (e.g., LGBTI) adults reporting lower loneliness, reduced stigmatization, increased opportunity for social and intimate relationships, greater openness about their identity, and greater overall contentment [62,63,64]. Friendships established within such communities often become a major source of fellowship and social support, in essence serving as a surrogate family [65,66].

### 1.5. Rationale for the Study

As noted, transgender individuals face significant challenges worldwide—including marginalization, isolation, and maltreatment—leading to feelings of isolation as well as to significant risk to their physical, psychological, and social well-being. For those gender/sexual minority individuals who find themselves socially marginalized or rejected (for whatever reason), the negative impact on their well-being can be mitigated through adaptive coping strategies and resilience [67,68].

Although the psychological effects of being transgender have been studied in more sexually open societies [68,69], relationships among coping strategies, feelings of loneliness, and QoL have been understudied in cultures in emerging/developing countries where openness and tolerance to diverse sexual identities are limited and where scarce health care resources are typically allocated to managing and controlling urgent infectious diseases. Nevertheless, expanded understanding of the relationships between coping strategies and QoL may assist human service providers in their support of transgender people living in communities by helping them develop the skills to deal with stress and loneliness and/or to replace maladaptive coping strategies with more positive adaptive strategies. Although re-integration into society-at-large is still unimaginable for transgenders in many cultures, better adjusted individuals may be more able to identify pathways to self-support and healthier life choices, reducing reliance on beggary and achieving higher QoL [9,70,71,72,73].

### 1.6. Aims of the Study

The aim of the present cross-sectional study, conducted from late 2018 to early 2020, was to explore the multidimensional relationships among loneliness, QoL, and coping strategies in a transgender population in Pakistan. As part of this study, we also wanted to verify relationships among these variables in a non-transgender group of similar social status and education, with the goal of understanding the benefit that various coping strategies bestow on transgender individuals living in community. To this end, we (1) assessed bivariate relationships among these variables in transgender and non-transgender groups; (2) assessed predictors of QoL based on loneliness, coping strategies, and gender status, as well as relevant demographic covariates; (3) determined whether specific coping strategies served as moderating variables to explain differences in loneliness and QoL between transgender and cisgender participants; and (4) assessed the role of coping strategies as a moderating variable between loneliness and QoL specifically within the transgender group.

## 2. Materials and Methods

### 2.1. Participants

Due to the challenges of recruiting transgender participants in Pakistan (see Section 2.3), purposive convenience sampling was used to enlist 200 participants from transgender communities in three major urban areas, Islamabad, Rawalpindi, and Faisalabad, with an estimated sample size determined using G * Power [74]. The term “transgender” in Pakistan reflects a mix of identity labels within a broad community of socially marginalized individuals [38] that include varying sexual and gender minority statuses (e.g., intersex, eunuchs, etc.). Inclusion criteria were self-identification as transgender, living in a transgender community for at least 1 year, 18 years or older, and having the ability to read Urdu (with or without assistance). Participation was limited to unemployed and/or having no formal occupation (thus, if employed, working in ad hoc/temporary positions for hourly wages), as these individuals represent the large majority and more vulnerable of those living in communities. Individuals from the community who were physically handicapped, had a major chronic disease, suffered from a diagnosed psychiatric illness, or were formally employed, were excluded.

A second group consisting of 100 heterosexual cisgender individuals was recruited to serve as a reference/comparison group in order to determine parallels in the role of social support in loneliness and/or QoL. (We considered this as a comparison/reference group rather than an actual control group.) Inclusion criteria included declared heterosexual cisgender status, 18 years or older, having the ability to read Urdu (with or without assistance), and unemployed and/or having no formal occupation (thus, if employed, working in ad hoc/temporary positions for hourly wages). In addition, we age-matched this group with the transgender group by limiting recruits to 20–58 years of age (the range for the transgender group). Because querying transgender individuals about birth sex is often considered inappropriate or taboo, cisgender participants were recruited to reflect the estimated proportion of (birth sex) male to female transgenders in the population. For this purpose, we relied on research on demographic trends in transgender identities suggesting a 2:1 male-to-female ratio [75], thus resulting in a total of 67 males and 33 females.

### 2.2. Assessment Instruments

#### 2.2.1. Demographic Questionnaire

This instrument collected information about age, education, occupation/work status, religion, income, and, when applicable, duration of living in the transgender community.

#### 2.2.2. UCLA Loneliness Scale (2013)

This validated (Cronbach α = 0.94) and reliable scale measures feelings of loneliness and social isolation [76]. Translated to Urdu [77], the scale consists of 20 items with response options on a 4-point scale. Items are summed to generate a global index ranging from 0 to 60. Higher scores indicate greater loneliness, and participants scoring 39 or higher are considered to have feelings of loneliness.

#### 2.2.3. WHO Quality of Life Bref (WHOQoL-BREF)

This cross-culturally validated (Cronbach α = 0.89) and reliable measure for assessing QoL [17], and translated into Urdu [78], consists of 26 items with each rated on a 5-point scale and generates scores on four domains: physical health, psychological health, social relations, and environment. Higher scores indicate higher QoL within each domain. More information can be obtained at https://depts.washington.edu/seaqol/docs/WHOQOL_Info.pdf (accessed on 15 June 2021).

#### 2.2.4. Brief COPE (2005)

Developed by Carver [22], an Urdu version [79] assesses participants’ coping strategies for everyday stressful situations. Brief COPE consists of 28 items rated on 4-point scales, yielding two subscales: adaptive coping and maladaptive coping. For each subscale, a higher score indicates greater use of that style. The scale has demonstrated reliability and validity (Cronbach α = 0.70) in a variety of populations [22,80].

### 2.3. Procedure

The recruitment of transgender persons (from transgender communities) in Pakistan is challenging, as such persons are largely invisible during much of the day, may have no stable living arrangement, and are often reluctant to participate in projects that might result in their being “outed.” They are most frequently found along roadsides and intersections engaging in beggary.

In recruiting participants, each transgender candidate was approached individually along the roadside and informed of the study’s purpose. The researcher’s reassurance of confidentiality and the potential value of the research, along with empathy and a non-judgmental demeanor helped allay the fears of many candidates who subsequently agreed to participate. If willing, the candidate provided informed consent as approved by the Higher Education Research Committee of Bahria University, Islamabad. Participants were assured anonymity, including the use of only aggregated data for research purposes. Participants then completed pencil-and-paper versions of the tests, with the investigator allowing each participant sufficient distance, space, and time to allow privacy so as not to influence responses. Upon request, the investigator aided those having difficulty reading or understanding the questions. When the respondent finished, the response form was inserted into a closed envelope. No individually identifying information was obtained from the respondent, either in-person or on the questionnaire.

A similar procedure was implemented for locating and recruiting cisgender participants. As with transgender recruits, many cisgender candidates occupied fixed spots along roadsides, waiting for someone to hire them for daily/temporary labor, vending, piecemeal work at home or in factories, and in various service establishments (salons, restaurants, etc.). Candidates were approached individually and asked if they would be interested in volunteering for a study on QoL. If so, details about the study’s purpose and procedures were explained, and for those who agreed to participate, the procedure followed that described for the transgender group.

### 2.4. Statistical Analysis

Data were analyzed using SPSS (IBM Corp. Released 2019. version 25.0. Armonk, NY, USA: IBM Corp.). Values for skewness for each of the variables of interest ranged from ±0.16 to ±0.65 (median = 0.39), well below the ±1.0 threshold generally requiring statistical adjustment. Bivariate correlations were used to establish relationships among predictor variables (Aim 1), as well as among subscales of the outcome variable (QoL). Forced entry linear regression analysis and moderating variable analysis [81] were used to predict QoL by sets of covariates (Aim 2) and to assess the potential moderating effect of coping strategies in (a) explaining differences between transgender and cisgender participants’ loneliness and QoL (Aim 3), and (b) explaining differences in QoL in just the transgender group (Aim 4).

## 3. Results

### 3.1. Description of Transgender and Cisgender Groups

Table 1 shows that the transgender group was slightly older and less educated than the cisgender group (*p* < 0.05), so these control covariates were included in the regression analyses. Table 2 shows descriptive statistics and the potential range of scores for the major study variables. Consistent with their marginalized status, the transgender group showed greater loneliness and lower QoL on three of four subscales (physical health, psychological health, and environment), but higher QoL on social relations.

### 3.2. Aim 1: Relationships among Measures

Table 3 provides Pearson correlations among predictor variables (loneliness and subscales of coping), and between predictor variables and QoL outcome variables. Predictor variables correlated significantly with all QoL outcome variables. QoL outcome variables also showed intercorrelations of 0.40 or higher, indicating these variables were, to some degree, tapping into the same broad construct of overall well-being. Correlations among variables were similar in direction for transgender and cisgender participants for all measures, with exceptions occurring only in age-related associations. Differences in the magnitude of associations among variables were also apparent for transgender vs. cisgender participants.

### 3.3. Aim 2: Predicting QoL with Variable Subsets through Regression

A composite outcome variable, “overall QoL”, representing the sum of the four QoL scales, was regressed on a set of variables that included loneliness, coping style (adaptive and maladaptive), group membership (trans vs. cis), and the non-collinear demographic variables of age and education. Significant predictors of higher QoL included younger age, lower loneliness, lower use of maladaptive coping strategies, and membership in the cisgender (vs. transgender) group (Table 4). Effect sizes as indicated by R^2^ values were large.

Regression analyses were also run separately on each of the four QoL subscales (physical health, psychological health, social relations, and environment) using the same set of predictor variables (Table 4). Higher physical health was predicted by lower age, lower maladaptive coping, and being cisgender. Higher psychological health was predicted by lower loneliness, lower maladaptive coping, and being cisgender. Higher social relations was associated with lower loneliness and being transgender. Finally, better environment was predicted by lower loneliness, being cisgender, and, marginally, lower education.

### 3.4. Aim 3: Coping Style as a Moderating Variable between Gender Status and QoL

Based on the research literature, we tested the extent to which coping strategies (adaptive or maladaptive) moderate differences between transgender and cisgender participants in loneliness and overall QoL. This procedure uses a two-step regression analysis in which the first model is run without the interaction term, then the second model includes the interaction term so as to determine whether the change in explained variance (R^2^) is significant [66].

For loneliness, both adaptive and maladaptive coping emerged as significant moderating variables between gender status (transgender vs. cisgender) and loneliness. Lower levels of adaptive coping and higher levels of maladaptive coping styles in the transgender group accounted for the greater loneliness in transgender participants (*p* ≤ 0.001). The change in explained variance, however, was small, 1.2% and 1.8%, respectively (*p* ≤ 0.001).

Neither adaptive nor maladaptive coping strategy moderated overall QoL, but both were significant moderators of the QoL subscales of physical health and social relations (Table 5). Specifically, greater maladaptive coping and lower adaptive coping in the transgender group were significantly associated with lower physical health (although the change in explained variance was under 1%, *p* = 0.038 and <0.001, respectively). However, greater adaptive coping and lower maladaptive coping in the transgender group were significantly associated with higher social relations, with the increase in explained variance of 4.5% and 4.6%, respectively (*p* < 0.001 for each).

### 3.5. Aim 4: Post Hoc Analyses Investigating the Transgender Group

#### 3.5.1. Aim 4a: Factors Predicting Loneliness and QoL

Within the transgender group, we determined the extent to which coping style, education, and duration in the transgender community predicted loneliness and QoL. Higher maladaptive coping, lower adaptive coping, higher education, and longer duration in the transgender community were associated with greater loneliness.

Overall QoL was significantly predicted by this same set of variables (Table 6). For QoL subscales, (1) better physical health was predicted only by shorter duration in the community; (2) better psychological health was predicted by lower maladaptive coping, higher adaptive coping, lower education, and longer time in the community; (3) better social relations was predicted by lower maladaptive coping, higher adaptive coping, and longer duration in the community; and (4) better environment was predicted by lower maladaptive coping, higher adaptive coping, lower education, and longer duration in the community. Effect sizes as indicated by R^2^ values were large.

#### 3.5.2. Aim 4b: Coping Styles as Moderating Variables in the Relationship between Loneliness and QoL within the Transgender Group

A moderating role for coping strategies was explored specifically within the transgender group in the relationship between loneliness and overall QoL (Table 7). Maladaptive coping explained 2% additional variance, and adaptive coping explained 3% additional variance in the relationship between loneliness and overall QoL (*p* < 0.001 for each).

For the QoL subscales, maladaptive coping explained 5% additional variance in the relationship between loneliness and physical health, 2% additional variance between loneliness and social relations, and 4% additional variance between loneliness and environment (*p* < 0.001 for each). Adaptive coping explained 16% additional variance in the relationship between loneliness and physical health (*p* < 0.001), 1% additional variance between loneliness and social relations (*p* < 0.01), and 1% additional variance between loneliness and environment (*p* < 0.001).

## 4. Discussion

To our knowledge, this is the first comprehensive study to investigate the role of coping strategies in predicting loneliness and QoL in a non-Western/South Asian sample of transgender individuals. Herein we demonstrated (1) that younger age, lower loneliness, lower use of maladaptive coping strategies, and membership in the cisgender (vs. transgender) group predicted higher overall QoL; (2) that combinations of variables including lower age, lower maladaptive coping, lower loneliness, and being cisgender predicted higher QoL subscale scores for physical, psychological, and environment domains; in contrast, being transgender predicted higher subscale scores for social relations; (3) that differences in adaptive and maladaptive coping styles between transgender and cisgender groups explained differences on the specific QoL subscales of physical health (lower in transgender participants) and social relations (higher in transgender participants); and (4) that within just the transgender group, lower maladaptive coping, higher adaptive coping, lower education, and longer duration in the community were significantly associated with higher QoL. In addition, for the transgender group, maladaptive and adaptive coping styles interacted with loneliness levels to explain QoL differences.

### 4.1. Predicting QoL in the Overall Sample of Transgender and Cisgender Individuals

#### 4.1.1. Loneliness as a Predictor

Independent of gender status, coping styles, loneliness, and QoL were all interrelated, such that lower loneliness (along with higher adaptive and lower maladaptive coping) predicted higher QoL. The general finding that loneliness was greater and QoL was lower in the transgender sample in Pakistan is consistent with studies demonstrating similar patterns in other geo-cultural regions [42].

Greater loneliness also strongly predicted QoL subscale scores: lower psychological health, lower social relations, and lower environment quality. The relationship of loneliness to psychological health has been documented in other populations (e.g., [72,73]), and regarding social relations, improving social networks and close relationships decreases loneliness, and this in turn can improve QoL and the person’s perception of their general environment/situation (e.g., [82,83]). Perhaps most striking in our study was that even though overall QoL and three subscales were lower in the transgender group, QoL related to social relations was higher—in our view, suggesting the compensatory impact of living within a supportive community for transgender individuals.

#### 4.1.2. Coping Skills as a Predictor

How individuals deal with personal and environmental stressors, including loneliness, is related to their perceived QoL. Such associations fit well with the minority stress model [35], which recognizes that the multiple stressors impinging on marginalized groups (including those in gender minorities) may not only negatively impact their ability to cope, but may also diminish their health-protective behaviors and increase their health-averse behaviors, leading to a cycle of deteriorating health, diminished sense of well-being, and increased risk for victimization and exploitation [35,36,37].

Loneliness in our sample (and elsewhere) was positively correlated with maladaptive coping—strategies that range from denial and withdrawal to high-risk activities (e.g., drug abuse, sex work, etc.), the latter having the effect of numbing or distracting from the loneliness, or providing personal affirmation from alternative group membership [84]. Further, maladaptive coping in our overall sample and, more specifically, in the transgender group, was associated with poorer physical and psychological health. Adaptive coping strategies, on the other hand, that include problem solving, developing self-help strategies, seeking social support, and relating to a peer group generally increased QoL. Although coping strategies may include seeking solace from within the environment, for example, by increasing one’s work burden or acquiring a hobby, such options are generally not available to transgender individuals in Pakistan, often unemployed and having access to few resources.

### 4.2. Coping Style as a Moderating Effect between Gender Status and QoL

Differences in coping styles between transgender and cisgender groups accounted, in part, for the differences in loneliness and in the QoL subscales of physical health and social relations. The negative effect of maladaptive coping on physical health in transgender participants is likely related to behaviors that impart greater health risks, such as drug use, sex work, aggression, and avoidance behaviors (e.g., not seeking needed medical help). Given that transgender individuals have fewer positive coping skill options, it is not surprising that they turn to more detrimental coping behaviors which, in turn, negatively affect their physical health.

Quite the opposite occurs with respect to social relations, where transgender individuals living in communities likely experience a benefit over non-transgender individuals. Specifically, faced with loneliness due to societal marginalization or family rejection, transgender people can seek out support communities that act as surrogate families and that offer social support, fellowship, shelter, and safety. Cisgender individuals of comparable social status and having little or no leisure time, by contrast, have few options to join supportive communities that could improve social connectedness and mitigate loneliness.

### 4.3. Understanding the Transgender Group

An important goal of this study was to document predictors of QoL specifically within the transgender population in Pakistan, and to explore whether differences in coping styles within this population moderated the relationship between loneliness and QoL. Specifically, coping styles (adaptive and maladaptive) played a strong and consistent role in overall QoL and subscales related to psychological health, social relations, and environment, following expected patterns that more maladaptive coping strategies lead to lower QoL, and more adaptive coping strategies lead to higher QoL. Interestingly, more education was associated with lower QoL overall and on the psychological health and environment subscales (although coping style was not education-related). We surmise that given the limited employment opportunities for transgender people in Pakistan, more educated transgender individuals experience frustration related to the disparity between their educational preparation and their highly restricted career options. Longer duration of living in the transgender community was also related to higher QoL overall; presumably, the longer the transgender person lived within the community, the deeper/better the social interactions, sense of family, psychological health, and overall perception of their environment.

Differences in coping strategies within the transgender group played important and strong moderating roles between loneliness and QoL, explaining anywhere from 1–16% additional variance in the model. For example, coping styles in response to loneliness explained large variances in physical health (see Supplementary Figure S1a,b), psychological health, and environment, with adaptive coping having a greater effect than maladaptive coping. That is, stronger adaptive coping skills mitigated the effects of loneliness on physical health, psychological health, and environment; conversely, higher maladaptive coping strategies worsened the negative effect of loneliness on these QoL indices. In essence, coping skills have a marked impact on how much or how little loneliness affects transgender QoL.

### 4.4. Interpretation, Implications, and Intervention

Depending on socio-cultural traditions and values, the attitudes of people toward the transgender community range from acceptance and curiosity to discrimination, disgust, rejection, and assault/aggression [29,30]. The colonial and post-colonial landscape in South and Central Asia has created a system of societal stigma that relegates transgender people to the margins, resulting in family rejection, societal and institutional discrimination, and a life often characterized by beggary and diminished QoL [47,48,49,51]. Negative attitudes toward transgender individuals are not specific to emerging/developing nations but are also found within cultures having greater openness to sexual diversity [25]. Indeed, some emerging nations (e.g., those having a pre-colonial tradition of non-binary classifications) have made significant legal strides to protect the rights of transgender people [85,86,87,88]. For example, giving a nod to its pre-colonial past, Pakistan’s Supreme Court has recently provided official recognition to the “third” gender in the citizen registration category, a recognition that affords both voting rights and access to health care [47,49].

Despite such progress, legal protection and equal rights continue to evade most transgender individuals; and discrimination, stigmatization, family humiliation, and marginalization persist in most cultures [56]. Such is the case in Pakistan where transgender individuals may be refused employment and deprived of educational opportunities, have limited access to basic physical and psychological support services; and continue to be objects of negative judgment and ridicule (considered immoral, deviant, and cursed) by the general populace [56,89,90]. Although transgender individuals from the upper classes may remain in their families—able to afford costly treatment and pursue a career—for most transgender individuals, traditional coping strategies such as pursuing employment, assimilating into mainstream society, and seeking financial and emotional support from friends and family are not viable options. Against this backdrop, many transgender people in Pakistan join transgender communities [90,91] because they are shunned, disowned, or even abandoned by their families, falling into poverty, unable to afford treatment, and otherwise doomed to a marginalized life. In doing so, they are able to access resources through communal beggary, offset loneliness, and improve their QoL [92].

Three findings from this study especially offer promise to transgender individuals in places such as Pakistan. First, as noted above, transgender and cisgender groups did not differ all that much in their adaptive coping strategies, only in maladaptive coping strategies. Second, QoL differences were related more to variance in coping strategies within the transgender group than to variance in coping strategies across transgender and cisgender groups. Third, increased positive and reduced negative coping strategies are associated with lower loneliness and higher QoL, suggesting the profound need for developing more effective coping strategies in transgender individuals. Such findings offer both insight and direction for interventions for transgender people living in geo-cultural regions where loneliness and low QoL result from family and societal marginalization.

Specifically, QoL and loneliness might be addressed through two complementary strategies, including (1) at the level of the individual, identifying, guiding, and encouraging the use of more positive/adaptive coping strategies and less maladaptive coping; and (2) at the level of the transgender community, identifying ways to optimize the support role it offers to members. Regarding the individual level, intervention might emphasize helpful information and behavioral/ psychological strategies for identifying and developing positive coping strategies—building, negotiating, and sustaining social relationships both within and outside the transgender community—and avoiding counterproductive strategies such as wishful thinking, avoidance/escape behaviors, self/other blaming, social withdrawal, and so on.

At the community level, human service professionals might help transgender communities take steps to maximize the support they provide to members by fostering positive and interpersonal dynamics among members and developing optimal organizational structures that specifically address each of the QoL subscales of physical health, psychological health, social relations, and environment. These professionals, in building trust with the transgender communities, could also assist community members in gaining access to needed services (housing, clinics) and could serve as experts/advocates to the outside world in matters related to policy, law, and human rights.

### 4.5. Limitations

A major limitation of this study was its cross-sectional nature, thereby preventing causal interpretations among loneliness, coping strategies, and QoL outcomes. Future research that implements intervention strategies at both the individual and transgender community levels, as described above, could help establish needed cause-effect relationships, while concomitantly offering valuable well-being services to transgender individuals and communities. We further recognize that our study focused on a specific group of avowed transgenders—those living in communities, lacking formal employment, and engaging in communal beggary. Thus, our results may not apply to transgender individuals who live with their families, have gainful employment, or have access to resources to overcome the obstacles to a better QoL. Additionally, we were unable to incorporate a rigidly defined control group: for example, transgender individuals not living in a community (such individuals represent a hidden group and would be impossible to identify), or cisgender individuals who were equated with transgender participants on predictors of loneliness. Finally, we recognize that the moderating effects tested in this study, though respectable by publication standards [93], were relatively small. Where possible, future research might incorporate better controls, assess the effects of intervention strategies on loneliness and QoL, and consider in-depth qualitative study not only of transgender individuals living in communities, but also of the transgender communities themselves so as to assist in understanding community characteristics that impart the greatest benefits to their members.

## 5. Conclusions

Transgender and cisgender groups do not differ all that much in their adaptive coping strategies, only in maladaptive coping strategies. QoL differences were related more to variance in coping strategies within the transgender group than to variance in coping strategies across transgender and cisgender groups, that is, coping skills have a marked impact on how much or how little loneliness affects transgender QoL. Given that increased positive and reduced negative coping strategies are associated with lower loneliness and higher QoL, there is a profound need for developing more effective coping strategies in transgender individuals. 

## Figures and Tables

**Table 1:** Means (±SE) and percentages (for categorical variables) of the demographic characteristics of sample (*N* = 300) and transgender and cisgender subsamples.

Participants Characteristics		Overall (*n* = 300)	Transgender (*n* = 200)	Cisgender (*n* = 100)	*p*-Value ^1^
		M (SE) or %	M (SE) or %	M (SE) or %	
Age (in years)		37.2 (0.47)	37.9 (0.56)	35.9 (0.83)	0.048
Education	<High School	48.3%	60.5%	24.0%	0.000
	≥High school	51.7%	39.5%	76.0%	
Religion	Muslim	80.0%	76.5%	87.0%	0.021
	Non-Muslim	20.0%	23.5%	13.0%	
Monthly Income (PKR)	≤15,000 ($100)	58.7%	47.0%	82.0%	0.000
	15,000–30,000 ($100–200 US)	41.3%	53.0%	18.0%	
Years in Community		NA	20.5 (0.54)	NA	

Note: ^1^ Comparisons were made with the *t*-test or chi square test.

**Table 2:** Means and SD for overall sample, transgender sample (*N* = 200), and cisgender sample (*N* = 100) for major study variables. Cronbach values are for the overall sample.

	No of Items	α	Range	Overall		Transgender		Cisgender		*p*-Value ^1^
			Potential	M	SD	M	SD	M	SD	
UCLA Loneliness Scale	20	0.88	0−60	44.93	7.07	47.56	5.99	39.67	6.09	<0.001
Brief COPE										
Maladaptive Coping	10	0.89	10−40	30.62	5.84	32.26	4.22	27.35	7.13	<0.001
Adaptive Coping	18	0.92	18−72	35.22	8.45	34.52	6.47	36.62	11.34	0.088
WHO Quality Life Brief										
Physical Health	7	0.88	7−35	18.80	5.54	15.46	2.18	25.49	3.94	<0.001
Psychological Health	6	0.90	6−30	12.56	5.10	9.42	2.11	18.83	3.15	<0.001
Social Relation	3	0.74	3−15	10.34	2.03	10.89	2.03	9.23	1.53	<0.001
Environment	8	0.79	8−40	16.19	6.64	11.76	1.52	25.04	3.15	<0.001

Note: M = Mean; SD = Standard deviation. ^1^ Comparisons were made between transgender and cisgender groups using the *t*-test.

**Table 3:** Pearson correlations among UCLA Loneliness Scale, Brief COPE scale (maladative and adaptive coping), and subscales of WHO Quality of Life for the transgender (T: *N* = 200) and cisgender (C: *N* = 100) subsamples.

Variable	2	3	4	5	6	7	8	9
1. UCLA Loneliness	(T) 0.65 ** (C) 0.81 **	(T) −0.66 ** (C) −0.82 **	(T) −0.48 ** (C) −0.56 **	(T) −0.92 ** (C) −0.74 **	(T) −0.87 ** (C) −0.59 **	(T) −0.83 ** (C) −0.47 **	(T) 0.48 ** (C) 0.71 **	(T) 0.48 **
2. Maladapt Coping		(T) −0.67 ** (C) −0.97 **	(T) −0.18 * (C) −0.53 **	(T) −0.67 ** (C) −0.74 **	(T) −0.59 ** (C) −0.55 **	(T) −0.61 ** (C) −0.37 **	(T) 0.15 * (C) 0.65 **	(T) 0.18 **
3. Adaptive Coping			(T) 0.31 ** (C) 0.52 **	(T) 0.64 ** (C) 0.73 **	(T) 0.57 ** (C) 0.54 **	(T) 0.57 ** (C) 0.39 **	(T) 0.32 ** (C) −0.65 **	(T) 0.34 **
4. WHO Physical Health				(T) 0.40 ** (C) 0.70 **	(T) 0.51 ** (C) 0.52 **	(T) 0.45 ** (C) 0.62 **	(T) 0.91 ** (C) −0.75 **	(T) 0.80 **
5. WHO Psychol Health					(T) 0.83 ** (C) 0.71 **	(T) 0.83 ** (C) 0.59 **	(T) 0.38 ** (C) −0.66 **	(T) 0.37 **
6. WHO Social Relations						(T) 0.80 ** (C) 0.64 **	(T) 0.49 ** (C) −0.45 **	(T) 0.46 **
7. WHO Environment							(T) 0.42 ** (C) −0.41 **	(T) 0.41 **
8. Age								(T) 0.88 **
9. Years in Community								

* *p* < 0.05, ** *p* < 0.01.

**Table 4:** Regressions on overall QoL and four QoL subscales for transgender and cisgender participants combined.

	Overall	Physical	Psychological	Social Relations	Environment
**Variable**	t (*p*-Value)	t (*p*-Value)	t (*p*-Value)	t (*p*-Value)	t (*p*-Value)
Age	−8.51 (**<0.001**)	−18.46 (**<0.001**)	−1.07 (0.287)	−1.41 (0.158)	−1.27 (0.204)
Trans/cis gender	−32.93 (**<0.001**)	−32.18 (**<0.001**)	−30.17 (**<0.001**)	18.69 (**<0.001**)	−39.25 (**<0.001**)
Loneliness	−9.88 (**<0.001**)	0.21 (0.213)	−11.65 (**<0.001**)	−13.63 (**<0.001**)	−6.47 (**<0.001**)
Education	−1.24 (0.217)	−0.63 (0.529)	−1.11 (0.268)	0.52 (0.602)	−1.93 (0.054)
Maladaptive Coping	−2.44 (**0.015**)	−1.98 (**0.049**)	−3.48 (**0.001**)	−0.51 (0.609)	−1.09 (0.277)
Adaptive Coping	−0.33 (0.741)	−0.47 (0.642)	1.08 (0.281)	−1.58 (0.114)	−0.14 (0.891)
*Adjusted R* ^2^	*0.80*	*0.83*	*0.80*	*0.67*	*0.91*

Note: Significant *p*-values are in bold.

**Table 5:** Test of coping style as a moderating variable between gender status and QoL in the overall sample.

	Overall	Physical	Psychological	Soc Relation	Environment
**Moderating Variable**	ΔR^2^ (*p*-Value)	ΔR^2^ (*p*-Value)	ΔR^2^ (*p*-Value)	ΔR^2^ (*p*-Value)	ΔR^2^ (*p*-Value)
Adaptive Coping × Gender Status	0.0% (0.832)	0.3% (**0.038**)	0.0% (0.882)	4.6% (**<0.001**)	0.0% (0.345)
Maladaptive Coping × Gender Status	0.0% (0.490)	0.9% (**<0.001**)	0.0% (0.861)	4.5% (**<0.001**)	0.1% (0.184)

Note: Significant *p*-values are in bold.

**Table 6:** Regressions on overall QoL and four QoL subscales for only transgender participants.

	Overall	Physical	Psychological	Social Relations	Environment
**Variable**	t (*p*-Value)	t (*p*-Value)	t (*p*-Value)	t (*p*-Value)	t (*p*-Value)
Years in Community	8.71 (**<0.001**)	−14.91 (**<0.001**)	2.78 (**0.006**)	4.70 (**<0.001**)	3.74 (**<0.001**)
Education	−3.54 (**0.001**)	−0.82 (0.414)	−4.09 (**<0.001**)	−1.95 (0.053)	−4.52 (**<0.001**)
Maladaptive Coping	−5.92 (**<0.001**)	0.25 (0.802)	−6.99 (**<0.001**)	−5.47 (**<0.001**)	−6.45 (**<0.001**)
Adaptive Coping	3.54 (**0.001**)	0.91 (0.362)	4.20 (**<0.001**)	3.03 (**0.003**)	2.73 (**<0.001**)
*Adjusted R* ^2^	*0.63*	*0.58*	*0.57*	*0.47*	*0.52*

Note: Significant *p*-values are in bold.

**Table 7:** Test of coping style as a moderating variable between loneliness and QoL in the transgender group.

	Overall	Physical	Psychological	Soc Relation	Environment
**Moderating Variable**	ΔR^2^ (*p*-Value)	ΔR^2^ (*p*-Value)	ΔR^2^ (*p*-Value)	ΔR^2^ (*p*-Value)	ΔR^2^ (*p*-Value)
Adaptive Coping × Loneliness	3.0% (**<0.001**)	16.0% (**<0.001**)	0.6% (**0.005**)	1.0% (**0.008**)	1.0% (**0.001**)
Maladaptive Coping × Loneliness	2.0% (**<0.001**)	5.0% (**<0.001**)	0.8% (**0.005**)	2.0% (**<0.001**)	4.1% (**<0.001**)

Note: Significant *p*-values are in bold.

## Data Availability

The datafile can be made available upon reasonable request to the first author.

## Supplementary Materials

The following are available online at https://www.mdpi.com/article/10.3390/ijerph18179109/s1, Figure S1a: The Relationship between loneliness and physical health is moderated by adaptive coping, Figure S1b: The Relationship between loneliness and physical health is moderated by maladaptive coping.
