# Supplementary material for: The Critical Role of Coping Strategies in Moderating Loneliness and Quality of Life: Parallel and Unique Processes among Transgender and Heterosexual Cisgender People in Pakistan
Source: Int J Environ Res Public Health. 2021 Aug 29;18(17):9109. doi: 10.3390/ijerph18179109 (PMC8431102; doi:10.3390/ijerph18179109)
Supplement: Supplementary file 1 [file ijerph-18-09109-s001.zip › ijerph-1283956-supplementary.pdf]

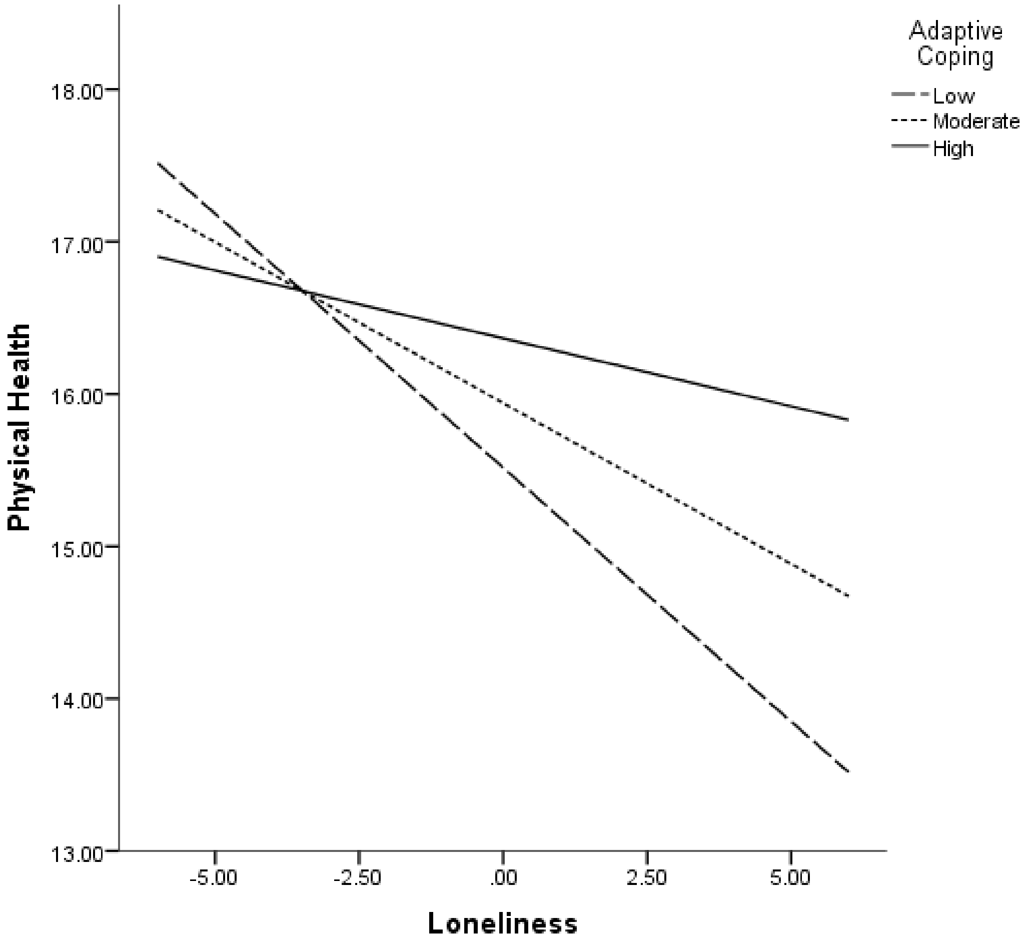

Figure S1a: The Relationship between loneliness and physical health is moderated by adaptive coping.

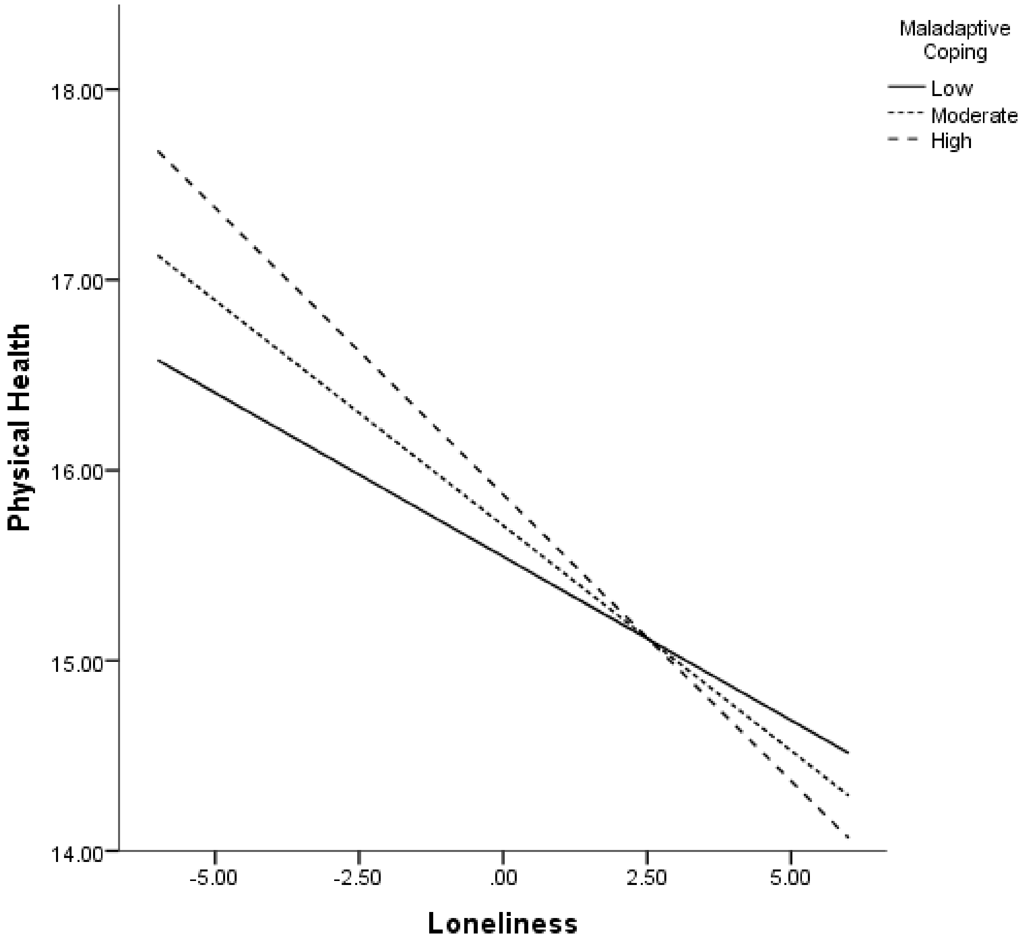

Figure S1b: The Relationship between loneliness and physical health is moderated by maladaptive coping.
